# Society Meetings

**Published:** 1910-06

**Authors:** 


### SOCIETY MEETINGS.

THE National Confederation of State Medical Examining and Licensing Boards will hold its twentieth annual meeting at St. Louis, Mo., on Monday, June 6, 1910, in the Southern Hotel.

The subjects to be taken up at this meeting will be a consideration of practical clinical instruction in medical colleges, a report on medical education in the United States by a representative of the Carnegie Foundation, and a report on a proposed materia medica list by a special committee. These topics are all practical and of vital interest to examining boards, medical schools and the profession. The contributors of papers to the symposium on clinical instruction are men of the highest standing in the medical profession, many of them teachers in some of the foremost institutions in this country, and their productions will be worthy of the most careful consideration. The chief object of this symposium is to determine, as far as possible, whether clinical instruction in medical schools can be made sufficiently practical and thorough, to warrant the medical boards in demanding practical examinations in the principal branches of the medical course.

An earnest and cordial invitation to this meeting is extended to all members of state boards, professors and teachers in medical schools, and all others interested in securing the best results in medical education. The officers of the Confederation are, President, A. Ravogli, M. D., 5 Garfield Place, Cincinnati, Ohio; Secretary, Murray Galt Motter, M. D., 1841 Summit Place, N. W., Washington, D. C.

---

THE American Gastroenterological Association will hold its thirteenth annual meeting at the Planters Hotel, Saint Louis, June 6 and 7, 1910.

---

THE American Academy of Medicine will hold its thirty-fifth annual meeting at the Hotel Jefferson, Saint Louis, Saturday, June 4 and Monday, June 6, 1910. Dr. James McBride, of Passadena, Cal., is the president, and Dr. Charles McIntire, of Easton, Pa., is the secretary.

---

THE Buffalo Academy of Medicine held meetings during the month of May, 1910, as follows:

*Section on Surgery.*—Tuesday evening, May 3. Program: The Symptomatology of Some Abdominal Lesions, Marshall Clinton.

*Section on Medicine.*—Tuesday evening, May 10. Program: (a) A Report on the Use of the *x*-rays and High Frequency Current in Treatment, A. W. Bayliss. (b) Meningitis, F. S. Crego. (c) Seeing and Feeling the Pulse, illustrated by lantern slides, Henry R. Hopkins.

*Section on Pathology.*—Tuesday evening, May 17. Program: Practical Clinical Consideration of Nephritis, Professor J. J. MacKenzie, M. D., Toronto, Canada.

*Section on Obstetrics.*—Monday evening, May 23. Program: Form Abnormalities and Complications of the Pregnant State with report of cases—specimens, L. G. Hanley; Some Thoughts on Retroversion, C. C. Frederick.

---

The Rochester Academy of Medicine held meetings during the months of April and May, 1910, at the Hotel Seneca, as follows:

*Section on Public Health.*—Wednesday evening, April 27. Program: Early Repair of the Cervix and Perineum, Evelyn Baldwin; report of cases.

*Section on General Medicine.*—Wednesday evening, May 4. Program: The Proposed Sewerage Plan for the City of Rochester, Assistant City Engineer, John F. Skinner.

*Section on Surgery.*—Wednesday evening, May 11, 1910. Program: Feet, Ralph R. Fitch.

*Section on Obstetrics.*—Wednesday evening, May 18. Program: Reports of Interesting Obstetrical Cases. Case reports will be made by Drs. O. E. Jones, Brown, Soble, McGill and Quigley.

